# The Old Doctor on “Embalmed Beef” and Things: Homeopathy to the Breach—A Story of a Cock and Bull

**Published:** 1899-02

**Authors:** 


### EDITORIAL DEPARTMENT.

F. E. DANIEL, M. D., Editor.
S. E. HUDSON, M. D., Managing Editor.
A. J. SMITH. M. D., Galveston, Associate Editor.

Published Monthly at Austin, Texas, by Drs. Daniel and Hudson. Subscription price $1.00 a year in advance.

Eastern Representative: John Guy Monihan, St. Paul Building, 220 Broadway New York City.

Official organ of the West Texas Medical Association, the Houston District Medical Association, the Austin District Medical Society, the Brazos Valley Medical Association, the Galveston County Medical Society, and several others.

### The Old Doctor on "Embalmed Beef" and Things: Homeopathy to the Breach—A Story of a Cock and Bull.

*"Its a very fine thing for to be a major-general."*—PIRATES OF PENZANCE.

"He *said* he'd prove it," said our Fat Philosopher, the genial old doctor, as honoring us today with a visit he dropped lazily into our easy chair, always vacated for him. "He *said* he'd prove it"!

"*Who* 'said he'd prove it'?" said I.

"Why Miles, The Gorgeous! Major-General Sir Nelson Alexander-the-great Miles, the 'Colossal,'" said the doctor.

"Prove what?" said I.

"Why, Dan'els, what ails ye? Don't you read the papers? The Gorgeous charged before the alleged 'committee of investigation' (he wasn't on oath, though) that the Commissary General of the U. S. Army had, as an experiment, issued to the soldiers beef impregnated with chemicals,—inferentially, poisonous chemicals,—and that it had caused the typhoid fever and other sickness in the army."

" Well," said I, "*has* he proved it?"

"Read this," said the doctor, handing me the following, clipped from the Philadelphia *Ledger* (January 28, ult.):

### WHAT A DOCTOR SAW.

### FLUID INJECTED INTO MEAT IN A BIG SLAUGHTERING ESTABLISHMENT.

**Dr. Christine Will Send to Major-General Miles an Affidavit Describing His Observations in Omaha.**

"Dr. G. Maxwell Christine, a well known homeopathic physician, said to a *Ledger* man last evening that he saw a process, at a large slaughtering establishment at Omaha, last June, that astonished him. He was a member of the Homeopathic Medical Association, which was holding its sessions in that city during the exposition period, and accepted an invitation tendered to the members to visit the great slaughter house. Everything there was done on a large scale, and each man had a particular part to do. To one of these was assigned the duty of thrusting a skewer into each piece of meat as it was passed rapidly by him. Pulling out the skewer, he smelt it, and quickly determined its condition.

"Another thrust into each piece of meat a canula, or steel skewer, with a hole running through it. This was attached to the end of a hose which supplied a fluid under pressure. A drip pan caught the fluid as the canula was pulled out of one piece and thrust into the next. It was a colorless liquid, the character of which Dr. Christine did not know. It was presumably a preserving liquid. Nor could he say that the meat was destined to be used in the army or navy.

"When the question was raised in Washington as to whether or not a preservative process had been used on the meat shipped for the use of the troops, he said, he believed it to be his duty to state the facts above narrated to the proper authorities, so that all the light possible might be thrown on the subject. He has communicated with General Miles, and, at the latter's request, will today forward an affidavit covering the above facts."

I read it. "Why, doctor," I said, "you don't call *that* evidence that General Eagan issued to the troops beef that had been impregnated with poisonous chemicals, and that he did it as an experiment, and that it gave the soldiers typhoid fever?"

"Never knew but one clearer case; it's 'confirmation strong as proof from Holy Writ,' " said the doctor.

"What was that other case, doctor?"

"Well it happened when I was a boy, going to school at an 'old-field school,'—girls and boys. One day after recess, Sissy Milligan went crying to the teacher, and sobbed: 'Billy Bones threw me down and took my—my—' well, you know; that precious possession of girlhood which once despoiled can never be restored. 'I didn't!' said Billy, stoutly, 'she's a "colossal—" ' 'Yessum he

*did,* Miss,' said Johnny Bright, 'cause I seen him runnin' 'round the corner of the house *chawin'* sump'n.' "

"Oh! doctor! doctor!" said I, hiding my face behind Hudson's blushes. (The office boy caught at a fly and pretended not to hear.)

"Fact," said the old doctor. After a moment he continued:

"Now, Dan'els, here's an alleged 'doctor' who goes to an alleged medical convention—ostensibly a gathering of medical scientists—and makes an alleged 'inspection' of a big beef-packing establishment. He sees a man stick a skewer in a piece of meat and smell it. He sees another inject a 'colorless fluid' in another piece, and pass it on. He doesn't ask what the 'colorless fluid' is, nor for what reason it is injected, and is not told. He does not know whether the beef was to be, or was later, furnished to the army or to the navy, or whether, being already 'spoilt,' it went to the soap pot. He came away as wise as he went; and now—he 'feels it his duty,' he 'presumes' that the 'colorless liquid' was a 'preserving fluid,' and General Miles is driven, or stoops, to use this rot to try to substantiate his infamous charges! But, Dan'els, an officer who would put irons on the captive Chieftain of a great and just (though 'lost') cause could be equal to doing anything mean, cowardly and contemptible."

" 'Embalmed,' in the sense intended, means 'impregnated with *drugs,* to prevent decomposition' (Webster). Chemicals? Yes, certainly. All meats are preserved, or 'cured,' by impregnating them with 'chemicals,' salt (chloride of sodium), and saltpetre (nitrate of potash) being those universally used. Hence, hams, shoulders and breakfast bacon, mess pork, and pig's feet, are 'embalmed' pork; corned beef is 'embalmed' beef. Among the tons of bacon furnished the army did the 'Georgeous' never hear of a cask or two that was 'spoilt'? Did he condemn the whole, therefore, as 'embalmed' with chemicals, and poisonous? or was the bad lot thrown back on the hands of the contractor, as the spoilt beef should have been? An unjust blow has been struck at one of our largest and most useful and important industries, and Miles has simply made himself ridiculous. Does any sane man suppose for a moment that 'capital' is going to invest in experiments? Not much. Money is the most timid thing in the world. To charge that those great capitalists would put their money into even a questionable process of curing meats is a stultification.

"The whole thing is so puerile as to look—to a man up a tree—very much like the fluttering of the old bird to lead away from its nest."

And the old doctor said he must be going, and he went.

A Fable (After Aesop).—A little Homeopathic Chicken, while playing under a Rose Bush, was Frightened by the falling of a Leaf. Being a Chick of Lively Imagination he ran piping to the Old Hen, and exclaimed: "Oh, hen-pen Miles, the sky's a' fallin' "! "How do you know, Chicken-little"? " 'Cause I saw it with my Eyes, I heard it with my Ears, and a part of it fell on my Tail."

(Old Hen, aside: "This is some of Eagan's work.) "Oh," said the Old Hen, "Let's go and tell the Old Gray Goose." And away they ran, with much Cackling, to the White House. "Oh Mother Goose," said the Old Hen, "the sky's a' fallin' "! "How do you know, Old Hen"? "Chicken-little told me." "How do you know, Chicken-little"? " 'Cause I saw it with my Eyes, I heard it with my Ears, and a part of it fell on my Tail"!

The Old Goose was much impressed. She Wept Copiously. Then, "mildly assimilating" a little Republican Policy (freshly made), as a bracer, she said: "Let's go and tell Mr. Sly-Fox; he's great at getting out of scrapes; got his tail caught in a Trap one day, but wriggled out,—some How." So, away they ran, with a Great Noise, down to the War Department. There they found the Old Fox in his Den, thoughtfully mixing some White Wash. "Oh, Mr. Fox, the sky's a fallin' "! "How do you know, Mother Goose"? "The Old Hen told me." "How do you know, Old Hen"? Chicken-little-Christine told *me*." "How do you know, Chicken-little"? " 'Cause I saw it with my Eyes, I heard it with my Ears, and a part of it fell on my Tail."

"All of you run in here," said the Old Fox. When they had done so, with a Sly Grin, he Bolted the Door. Letting the Goose and the Chick out the Back Way, he leisurely proceeded to Embalm the Old Hen, and lay her on the Shelf.

